# Treatment of Nonalcoholic Fatty Liver Disease (NAFLD) in patients with Type 2 Diabetes Mellitus

**DOI:** 10.1186/s40842-016-0027-7

**Published:** 2016-04-12

**Authors:** Paola Portillo-Sanchez, Kenneth Cusi

**Affiliations:** 1 Division of Endocrinology, Diabetes and Metabolism, University of Florida, 1600 SW Archer Road, room H-2, Gainesville, FL 32610 USA; 2 Division of Endocrinology, Diabetes, and Metabolism, Malcom Randall Veterans Affairs Medical Center, Gainesville, FL 32608 USA

**Keywords:** Nonalcoholic fatty liver disease (NAFLD), Nonalcoholic steatohepatitis (NASH), Type 2 diabetes mellitus, Insulin resistance, Metformin, Thiazolinediones, Pioglitazone, Dipeptidyl peptidase 4 (DPP-4) inhibitors, Glucagon-like peptide-1 receptor agonists (GLP-1RA), Sodium-glucose co-transporter 2 (SGLT2) inhibitors

## Abstract

Nonalcoholic fatty liver disease (NAFLD) is believed to be the most common chronic liver disease, affecting at least one-third of the population worldwide. The more aggressive form is known as nonalcoholic steatohepatitis (NASH) and characterized by hepatocyte necrosis and inflammation. The presence of fibrosis is not uncommon. Fibrosis indicates a more aggressive course and patients with NASH that are at high-risk of cirrhosis and premature mortality, as well as at increased risk of hepatocellular carcinoma (HCC). Patients with type 2 diabetes mellitus (T2DM) are at the highest risk for the development of NASH, even in the setting of normal plasma aminotransferase levels. The presence of dysfunctional adipose tissue in most overweight and obese subjects, combined with insulin resistance, hyperglycemia, and atherogenic dyslipidemia, contribute to their increased cardiovascular risk. Many therapeutic agents have been tested for the treatment of NASH but few studies have focused in patients with T2DM. At the present moment, the only FDA-approved agents that in controlled studies have shown to significantly improve liver histology in patients with diabetes are pioglitazone and liraglutide. Current research efforts are centering on the mechanisms for intrahepatic triglyceride accumulation and for the development of steatohepatitis, the role of mitochondrial dysfunction in NASH, and the impact of improving glycemic control *per se* on the natural history of the disease. This brief review summarizes our current knowledge on the pharmacological agents available for the treatment of NASH to assist healthcare providers in the management of these challenging patients.

## Background

Nonalcoholic fatty liver disease (NAFLD) is the most common liver disease in the United States [1], affecting about 60–70 % of all obese individuals. With one-third of adults being obese, and a similar proportion being overweight, a large number of Americans are at risk of developing the disease [2]. The future magnitude of the problem can be better appreciated knowing that more than one-third of the pediatric population is overweight or obese [3]. Nonalcoholic fatty liver disease encompasses a broad spectrum of disease severity, ranging from isolated steatosis to its more severe form with variable degrees of hepatocyte inflammation, necrosis and liver fibrosis, known as nonalcoholic steatohepatitis (NASH) [4, 5]. Of note, among the histologic features of NASH, fibrosis is the more strongly correlated with end-stage liver disease and increased mortality [6, 7]. The presence of obesity and insulin resistance, often with clinical features of the metabolic syndrome (but not necessarily), leads to a high-risk profile for the development of NAFLD. However, it is the presence of T2DM that confers the highest risk for NAFLD, and specially, for NASH. The highest mortality in NAFLD/NASH arises not from end-stage liver disease, or an increased risk of hepatocellular carcinoma (HCC), but secondary to cardiovascular disease [8, 9]. This may be explained by worse atherogenic risk factors such as hyperglycemia, hyperinsulinemia [10] and dyslipidemia [11], among others.

**Table 1:** Therapeutic agents for T2DM and their effect NAFLD/NASH in clinical trials

Treatment	Mechanism of action	AST/ALT	Liver fat by imaging	Liver histology
Oral				
Metformin [38,45-48]	Insulin-sensitizer	↓	↓*,↔^	Unchanged
Pioglitazone [52, 53, 55]	PPARγ agonist	↓	↓^	Improved
Sitagliptin [72, 80, 81]	DPP-4 inhibitor	↓	n/a	n/a
Vildagliptin [82]	DPP-4 inhibitor	↓	↓^	n/a
Canagliflozin [90]	Inhibits renal glucose reabsortion	↓	n/a	n/a
Dapagliflozin [91, 92]	Inhibits renal glucose reabsortion	↓	n/a	n/a
Injectable				
Exenatide [70]	GLP-1 receptor agonist	↓	↓^	n/a
Liraglutide [69-75]	GLP-1 receptor agonist	↓	↓**^,^^	Improved

*NAFLD assessed by ultrasound, **NAFLD assessed by CT, ^NAFLD assessed by MRI/^1^H-MRS, n/a: data not available

Unfortunately, in the clinical setting, the lack of a reliable non-invasive diagnostic blood test or imaging technique for its diagnosis has let to underestimate the true prevalence of this condition. The prevalence of NAFLD in the general population is 34 % measured by the gold-standard technique of magnetic resonance and spectroscopy (^1^H-MRS), but about 2-fold higher in obese patients [12]. In a recent study in obese patients with T2DM and normal liver aminotransferases, the prevalence of NAFLD was 56 % and more than half of those undergoing a liver biopsy had NASH [13]. A population-wide liver biopsy study examining the prevalence of NASH in T2DM is not available given the invasive nature of the procedure. However, in a recent study in 3041 patients from the Rotterdam Study, a population-based study among individuals ≥45 years of age, Koehler et al. [14] reported that one out of six patients with T2DM had a diagnosis of fibrosis using a combined non-invasive screening approach of liver ultrasound and liver transient elastography. Taken together, it has become evident that NASH is a significant risk factor for future severe liver and cardiovascular disease. There is a need for increased awareness among clinicians to diagnose and treat early-on these patients.

## Role of dysfunctional adipose tissue and lipotoxicity

The pathophysiology of NAFLD is complex and multifactorial [1, 15, 16]. In the context of obesity and T2DM (both highly prevalent in NAFLD), mitochondrial dysfunction, insulin resistance and adipose tissue inflammation play a major role. It is well established that white adipose tissue is an active endocrine organ with potential to have major metabolic effects [17]. Adipose tissue secretes a diversity of adipokines that can have a pro- or anti-inflammatory effects [18, 19]. Obesity is commonly linked with adipose tissue activation of macrophages that promote a proinflammatory state and a state of subclinical inflammation that is also typical in patients with NAFLD. The presence of dysfunctional adipose tissue leads to increased rates of lipolysis and flux of free fatty acids to ectopic tissues activating apoptotic pathways and/or generating subsequent insulin resistance in muscle and liver [20]. The development of a “lipotoxic state” in NASH is central to activation of inflammatory pathways that lead to hepatocyte necroinflammation, and potentially a major target for therapy, as will be discussed in the next section.

## Therapeutic interventions

### Diet and lifestyle modification

Many studies have been done in NAFLD involving lifestyle modification and/or weight loss by dietary modification only. Most studies report a reduction in plasma aminotransferases and often improvement in hepatic steatosis. However, the data is often from small, poorly controlled studies and have rarely focused on patients with T2DM. Also, most have used surrogate endpoints to assess liver improvement, such as plasma aminotransferases or liver ultrasound, and rarely liver histology [21, 22]. A number of recent studies quantifying hepatic steatosis by ^1^H-MRS indicate that a modest weight reduction in the range of 5–10 % with exercise alone, or combined with caloric restriction, significantly reduces intrahepatic triglycerides (IHTG) by about 40 % [20]. In general, reduction in IHTG content (and improvement in cardiovascular risk factors) is proportional to the magnitude of weight loss induced by lifestyle intervention, as well demonstrated in a substudy of the Look Ahead that quantified hepatic triglycerides by ^1^H-MRS [23]. For instance, patients losing 5 to 10 % of weight had a 65 % decrease in IHTG, while the few achieving a ≥10 % lost 80 %. Similar results were reported by Wong et al. [24] after lifestyle intervention in a 12-month RCT in 154 patients with NAFLD, where a significant 5.6 kg total body weight reduction was associated with a 55 % decrease in IHTG (liver triglyceride content changing from 12.3 to 5.5 %). Unfortunately, the impact on liver histology was not examined in either study, but Promrat et al. [25] did report that a reduction of total body weight of ≥9 % is associated with a broad histological improvement in steatosis, necrosis and inflammation in patients with NASH. Patients with NASH undergoing bariatric surgery also have in most cases a reduction of hepatic steatosis, hepatocyte necrosis and inflammation, with less certain effects on fibrosis [26, 27]. However, improvement in fibrosis by weight loss or lifestyle changes is less well established and no specific dietary plan (other than caloric restriction) appears to be uniquely beneficial in NASH. The weight loss agents phentermine/topiramate, lorcaserin and naltrexone/bupropion have not undergone careful testing in a RCT in patients with NASH, but they may likely assist in improving histology as they may decrease body weight by ~10 % at maximal doses. In any case, improvement in dietary habits and increased physical activity leading to weight loss are fundamental interventions for the treatment of both T2DM and NAFLD.

### Pharmacological agents

Many agents have been tested for the treatment of NASH but most not specifically in patients with T2DM. For instance, agents that modify cholesterol metabolism such as statins or ezetemibe have been extensively studied [28, 29]. Statins can be safely given to patients with NAFLD/NASH to reduce their increased cardiovascular risk [30]. Several small studies have suggested some benefit of statins in NAFLD/NASH, although usually they have been short-term, uncontrolled trials using surrogate primary end points (such as plasma aminotransferases or imaging) rather than liver histology. No histological improvement was reported when comparing statin therapy versus placebo in the only 12-month controlled study using liver histology as the primary endpoint [31]. Taken together, there is consensus that statins do not improve hepatocyte ballooning or fibrosis in NASH, but are safe to prevent cardiovascular disease in this population. Ezetimibe may decrease IHTG, but its efficacy to improve steatohepatitis is unknown [32].

Omega-3 polyunsaturated fatty acids (PUFAs) activate peroxisome proliferator-activated receptor (PPAR) α receptors, which upregulate several genes involved in fatty acid oxidation, holding potential to reduce hepatic steatosis and downregulate pro-inflammatory pathways in NAFLD/NASH. While small, uncontrolled studies have reported a reduction in plasma aminotransferases and liver steatosis, recent studies with omega-3 fatty acids have been negative [33–36]. In summary, there appears to be no major role for PUFAs for the treatment of NASH.

Vitamin E is an anti-oxidant believed to reduce hepatocyte oxidative stress in patients with NASH [1]. In non-diabetic patients with biopsy-proven NASH vitamin E led to significant histological improvement in the primary endpoint (improvement in ≥2 grades in the NAFLD activity score [NAS], including hepatocellular ballooning and with no worsening of fibrosis), compared to placebo (*p* = 0.001) [37]. However, resolution of NASH did not reach statistical significance (36 % vs. 21 %, *p* = 0.05), an endpoint only reached in the same study by pioglitazone (47 %, *p* = 0.001 vs. placebo). Vitamin E also did not significantly improve histology in a large RCT in a pediatric population with NASH, although some histological features did improve (i.e., hepatocellular ballooning) [38]. In summary, vitamin E may be beneficial in patients with NASH without T2DM because it is relatively inexpensive and at the dose used (800 IU per day) it appears to be safe [39, 40]. However, its long-term efficacy has not been established in NASH and it has not been tested in patients with T2DM.

Pentoxifylline is a non-selective phosphodiesterase inhibitor that may decrease inflammatory pathways in NASH, such as TNF-α [28]. Several open-label, small studies reported mixed results on plasma aminotransferases and hepatic steatosis on imaging [41]. Two small 12-month studies have reported on histology. While in one study in 30 patients with NASH pentoxifylline did not improve histology compared to placebo [42], another small trial (*n* = 20 patients) reported some improvement in steatosis, lobular inflammation and fibrosis after 1 year [43]. More work is clearly needed to define its role in NAFLD and in patients with T2DM, a population never studied with this agent.

#### Metformin

Metformin is considered first-line therapy for the management in T2DM given its ability to improve insulin action and lower plasma glucose without hypoglycemia, as well as its potential for weight loss [44]. Given that patients with NAFLD/NASH have insulin resistance, it has been extensively tested in this setting. Early studies suggested histological benefit, but this was likely more related to concomitant weight loss in uncontrolled studies than to the biguanide *per se* [45, 46] (Table 1). For instance, in the study by Loomba et al. [45], the correlation of histological improvement with weight loss was very high (*r* = 0.79, *p* < 0.0001), and improvement in the NAFLD activity score happened largely in patients that lost ≥5 % of body weight (i.e., in four out of five patients). More recently, similar negative results were also reported in a RCT in 173 children (mean age: 13 years) assigned to either metformin 1000 mg, vitamin E 800 U or placebo for 96 months [38]. Several recent meta-analysis have included better-controlled studies and have concluded that metformin is not effective for the treatment of NASH [47, 48]. However, it must be highlighted that all glucose-lowering drugs approved for the treatment of type 2 diabetes are safe in NAFLD/NASH, unless advanced cirrhosis. Metformin is not liver-metabolized but largely eliminated by renal clearance, and can can be used safely in the vast majority of patients with NASH.

#### Thiazolinediones: role of pioglitazone

Thiazolinediones (TZDs) are ligands for the transcription factor PPAR-γ that plays a key role in the regulation of glucose and lipid metabolism, as well as in inflammation [49]. PPAR-γ is predominately expressed in adipose tissue, but is also present in many other tissues that regulate metabolic pathways such as the pancreas, liver and muscle. At least part of the mechanism of action of TZDs relates to restoring normal adipose tissue biology. Treatment with TZDs is associated with an amelioration of proinflammatory adipokines and an increase in the secretion of adiponectin by adipocytes [50], restoring the response of adipose tissue to insulin action, as well as at the level of the liver and skeletal tissue [20]. Evidence for the long-term impact of these broad metabolic and anti-inflammatory effects can be appreciated in the potential for pioglitazone to prevent the progression to T2DM in patients with impaired glucose tolerance, improving not only plasma glucose levels but also blood pressure and lipid levels [51], all factors that play an important role in the morbidity and mortality of patients with T2DM.

Both pioglitazone and rosiglitazone have been tested in patients with NASH [47]. Early studies were small, uncontrolled and reported mixed clinical results [20, 47] Belfort et al. [52], in a proof-of-concept 6-month trial in patients with prediabetes and T2DM, reported the first RCT of pioglitazone (plus a -500 kcal/day caloric restricted diet) in patients with biopsy-proven NASH. Patients had a significant improvement in hepatic steatosis, and necroinflammation. The NAFLD activity score (NAS) improved in 73 % of patients treated with pioglitazone compared to only 24 % in the placebo group with diet alone (*p* < 0.001). There was also a suggestion that fibrosis could be reversed in NASH, as pioglitazone-treated patients had a significant reduction in liver fibrosis compared to baseline, although this fell short of reaching statistical significance when compared to placebo (*p* = 0.08). This study was important in establishing that the histological abnormalities present in NASH could potentially be halted, and even reversed, within a relatively short period of time. Two additional RCTs later extended these findings to patients with NASH but without diabetes [37, 53], but rosiglitazone proved to be rather ineffective [54]. Recently, Cusi et al. [55] reported a 36-month study in 101 patients with prediabetes or T2DM and NASH. They found sustained histological and metabolic benefit with long-term treatment. Of note, there was also a modest but statistically significant difference in the mean scores for fibrosis when compared to placebo. The fact that improvement persisted over time suggests that pioglitazone may modify the natural history of the disease [56]. Treatment was well tolerated, with only mild weight gain (2.5 kg versus placebo). Potential side effects associated with TZDs include weight gain from either improved insulin action in adipocytes with increased triglyceride synthesis and overall adiposity, or rarely, from fluid retention. Development of shortness of breath or congestive heart failure is rare (~1 % or less) in the setting of pioglitazone treatment, and is related to unknown diastolic dysfunction that is common in T2DM. Finally, mild bone loss may occurr in women [49]. Recently, Lewis et al. [57] demonstrated in a long-term prospective study lack of an association between pioglitazone and bladder cancer. Of note, TZDs treatment has been associated with a reduction in a broad spectrum of cancers [58, 59] and with a decrease in cardiovascular mortality [60].

#### Glucagon-like peptide-1 (GLP-1) receptor agonists

GLP-1 receptor agonists (GLP-1RA) may become an attractive therapeutic option for the treatment of patients with T2DM and NASH given their broad spectrum of effects on glucose and lipid metabolism. Their structure prevents them from being immediately cleaved and inactivated by circulating dipeptidyl peptidase (DPP)-4 prolonging their systemic actions. Glucose-lowering by GLP-1RA is attributed to enhancement of insulin secretion, reduction of postprandial glucagon concentration, effects at the level of the central nervous system with appetite suppression and induction of weight loss, as well as improvement of insulin action in hepatocytes and adipose tissue [61]. It has been proposed that there may be direct binding to hepatic GLP-1 receptors that may account for at least part of the beneficial metabolic action in the liver [62–64]. A number of animal studies have shown that GLP-1 analogs improve hepatic insulin sensitivity and decrease steatosis [62, 64–66], and even fibrosis [67]. Exendin-4 significantly reduces hepatic de novo lipogenesis (DNL) in vitro and in vivo [68]. This is important as increased DNL is believed to play a significant role in NAFLD.

Several studies hinted at a potential benefit of GLP-1RA, but evidence until recently was inconclusive. A significant decrease in plasma aminotransferase levels and hepatic steatosis (assessed by CT scan) was observed at the higher dose of liraglutide (1.8 mg) in a meta-analysis of 6 RCT LEAD (Liraglutide Efficacy and Action in Diabetes) trials [69]. However, this difference was not significant after adjustment for weight (*p* = 0.25) or A1c (*p* = 0.93), suggesting an effect primarly associated with these factors. In an early study, Cuthberson et al. [70] found that 6 months of GLP-1RA therapy (19 with exenatide and 6 with liraglutide) was associated with significant weight loss of 5.0 kg, a 1.6 % A1c reduction and a 42 % relative reduction in IHTG (all *p* < 0.001). The relative reduction in IHTG correlated with A1c (*r* = 0.49; *p* = 0.01), but not significantly with change in total body weight, visceral or subcutaneous adipose tissue. Similar findings have been reported in other small uncontrolled studies [71, 72]. Eguchi et al. [73] also reported a reduction in body mass index (BMI), visceral fat accumulation, aminotransferases and hyperglycemia in 19 patients with biopsy-proven NASH treated with liraglutide for 24 weeks, with histological improvement in 6 out of 10 who had a repeat liver biopsy after 96 weeks of liraglutide treatment. Finally, in a recent uncontrolled study while both insulin glargine and liraglutide similarly decreased A1c levels, only insulin significanty decreased IHTG from baseline [74]. However, the study was hampered by the small sample size (*n* = 35) and significant number of dropouts (~20 %), short duration of follow-up (3 months) and the low-dose of liraglutide used (1.3 mg/day).

In the most comprehensive study to date, the LEAN (Liraglutide Efficacy and Action in Non-alcoholic steatohepatitis) trial showed benefit when 52 patients with biopsy-proven NASH were treated for 48 weeks with liraglutide at a dose of 1.8 mg per day [75]. Only one-third of the population had T2DM, but overall patients were obese and had evidence of insulin resistance. After treatment, 39 % of patients that received liraglutide had resolution of NASH (defined as disappearance of hepatocyte ballooning without worsening of fibrosis) compared to only 9 % in the placebo arm. While fibrosis did not improve with liraglutide, more patients in the placebo arm experienced worsening of fibrosis (*p* = 0.04). Patients treated with liraglutide had a significant reduction of body weight, fasting plasma glucose and A1c levels. A subset of patients accepted to undergo in-depth glucose and lipid turnover studies before and after 12 weeks of liraglutide treatment. This resulted in improved insulin sensitivity at the level of the liver, as well as a decrease in hepatic DNL. Insulin action in adipose tissue also improved, both fasting and in response to low-dose insulin infusion (i.e., suppression of systemic and with directly measured subcutaneaous microdialysis techniques). However, the improvements in insulin sensitivity were overall modest, and confounded by weight loss. Moreover, enhanced adipose tissue insulin sensitivity was not observed (i.e., Adipo-IR_index_) when the entire cohort was analyzed [75].

Taken together, treatment of patients with T2DM and NAFLD with GLP-1RA is an approach that calls for larger, long-term studies, to fully understand their therapeutic potential and the role of mechanisms that account for their efficacy beyond weight reduction or amelioration of hyperglycemia.

#### Dipeptidyl peptidase-4 (DPP-4) inhibitors

An alternate therapetic option for the treatment of NAFLD in T2DM are DPP-4 inhibitors. Their mechanism of action is primarily from the blockade of the multifunctional protein DPP-4 that degrades glucagon-like peptide-1 (GLP-1) and plays a key role in postprandial glucose homeostasis [76, 77]. These pharmacolgical agents primarily lower the postprandial plasma glucose (~50 mg/dl) and reduce A1c levels by ~0.6 to 0.8 % [78]. Also animal studies have shown their ability to reduce liver triglyceride content and inflammation [79].

The role of these agents in NAFLD has been tested in short-term clinical trials. Reduction in plasma aminotransferases have been reported with sitagliptin in Japanese patients with T2DM and NAFLD [72, 80]. In contrast, in another study plasma aminotransferases did not improve in patients with biopsy-proven NAFLD treated with sitagliptin for 12 months [81]. In a 6-month RCT in 44 patients with T2DM, Macauley et al. [82] reported that vildagliptin 50 mg twice daily significantly decreased plasma alanine aminotransferase (ALT) levels (from 27 to 20 IU/L, *p* < 0.001) and IHTG by ^1^H-MRS (from 7.3 % to 5.3 %, *p* = 0.001). Of note, plasma ALT was not truly elevated at baseline. Moreover, IHTG were only mildy increased before treatment (normal considered to be ≤5.5 %). However, a robust correlation was found between a decrease in the plasma aminotransferases and the triglyceride liver content (*r* = 0.83; *p* < 0.0001), confirming the close correlation between liver triglyceride content and plasma ALT [83]. In this study, there was no improvement in liver, muscle or adipose tissue insulin sensitivity. Reduction in IHTG did not correlate with changes in body weight but rather with the decrease in fasting plasma glucose at 3 months (*r* = 0.47; *p* = 0.02) and 6 months (*r* = 0.44; *p* = 0.03).

It should be noted that overall, the effect of DPP-4 inhibitors appears to be modest, although large RCTs with histological endpoints are lacking to conclusively establish their role in patients with T2DM and NASH.

#### Sodium-glucose co-transporter 2 (SGLT2) inhibitors

These agents inhibit the reabsorption of glucose in the proximal tubular system with a marked reduction of plasma glucose levels [84, 85]. At the present moment three agents are approved: dapagliflozin, canagliflozin and empagliflozin. Their use is also associated with a reduction of total body weight, possibly as a secondary effect of caloric loss and increased diuresis [86]. Recently, empagliflozin has been associated with a reduction in cardiovascular mortality [87].

Studies performed in rodents have shown that SGLT2 inhibitors, in addition to the reduction of plasma glucose concentration, decrease hepatic triglyceride accumulation and other inflammatory biomarkers [88, 89]. While these agents are now well-established therapeutic agents for the treatment of T2DM, their specific impact in NAFLD remains to be determined. The administration of canagliflozin (100 or 300 mg per day) for 52 weeks is associated with a reduction in plasma liver aminotransferases, especially at the higher dose, but their impact on liver histology is unknown [90]. While a small study did not see such en effect with dapagliflozin [91], Bailey et al. [92] reported a reduction in plasma ALT concentration in a larger 24-week trial. Considering that these pharmacologic agents ameliorate hyperglycemia, induce weight loss, and may improve insulin sensitivity, they are actively being explored for the treatment of NAFLD/NASH in patients with T2DM.

## Future treatments

Awareness about the high prevalence and significant health risks associated with NASH in patients with T2DM has led in recent times to a major drug discovery effort in this field. As a result, a variety of clinical trials are being conducted for the treatment of NASH, as reviewed elsewhere [93, 94]. For instance, the FLINT study found benefit with the treatment of obeticholic acid, a farnesoid X receptor (FXR), compared to placebo [95]. Recently, results from the GOLDEN 505 trail, a PPAR α/δ agonist, believed to combine the benefits on lipid metabolism of a PPARα and the effects on insulin sensitivity of PPARδ agonist, failed to meet the primary endpoint of resolution of NASH but reported some histological benefit in patients with a higher NAS score (≥4) [96].

## Conclusions

The presence of NASH is common among patients with T2DM, putting them at risk of end-stage liver disease, HCC and cardiovascular disease. Physicians must increase their awareness about the high-risk of complications associated with NAFLD/NASH and the emerging broad spectrum of therapeutic options. Lifestyle modification is essential. While a Mediterranean diet has been advocated to be beneficial, well-designed controlled studies are needed [97] and the best diet for the long-term management of NAFLD remains uncertain. Caloric restriction with weight loss ≥10 % appears to be essential for histological response. The addition of pharmacologic agents early-on, especially pioglitazone, has the potential to improve the quality of life and prevent severe comorbidities in this population.

## Abbreviations

^1^H-MRS: proton magnetic resonance spectroscopy
A1C: hemoglobin A1c
Adipo-IR: adipose tissue insulin resistance
ALT: alanine aminotransferase
BMI: body mass index
CT: computed tomography
CVD: cardiovascular disease
DNL: de novo lipogenesis
DPP-4: dipeptidyl peptidase-4
GLP-1RA: glucagon like peptide-1 receptor agonist
HCC: hepatocellular carcinoma
IHTG: intrahepatic triglycerides
NAFLD: nonalcoholic fatty liver disease
NAS: NAFLD activity score
NASH: nonalcoholic steatohepatitis
PPAR: peroxisome proliferator-activated receptor
PUFAs: polyunsaturated fatty acids
RCT: randomized controlled trials
SGLT2: sodium-glucose co-transporter 2
T2DM: type 2 diabetes mellitus
TZDs: thiazolinediones
